# A Comparative Study to Evaluate the Efficacy of Topical and Infiltrative Anesthesia for Dermatosurgery Through a Subject-Oriented Approach

**DOI:** 10.7759/cureus.57966

**Published:** 2024-04-10

**Authors:** Preetham Pottipati, Ganesa Sooria Kathirvel, Selva Sudha M, Dhanalakshmi Kathirvel

**Affiliations:** 1 Dermatology, Venereology and Leprosy, Karpaga Vinayaga Institute of Medical Sciences and Research Centre, Chengalpattu, IND

**Keywords:** alkalinized lignocaine 2%, aesthetic dermatology, dermato-surgery, eutectic mixture of local anesthesia, topical anesthetics

## Abstract

Introduction

The skin, along with its subcutaneous tissue, constitutes one of the major organ systems of the human body. Dermatosurgery is the branch dealing with skin conditions that operate at the level of skin, without disturbing the *milieu intérieur* of the human organ system.

Materials and methods

A cross-sectional study was undertaken, which included 100 patients comprised of 50 patients in each group. For group A, the topical anesthetic agent used was a eutectic mixture of topical 2.5% lignocaine and 2.5% prilocaine cream (EMLA); for group B, infiltration anesthesia with 2% lignocaine injection. Patients satisfying the inclusion and exclusion criteria were recruited for the study. All the participants were requested to rate the pain at the time of drug administration, during the dermatosurgical procedure, and post-procedurally with a visual analogue scale (VAS) separately.

Results

In this study, 50% of the participants were of the age group of 21-40 years. Males constituted 57.8% whereas females constituted 42.2%. The common procedures performed in the study were electrocautery at 37%, intralesional platelet-rich plasma (PRP) at 16%, and intralesional steroid at 7%. In group A, the VAS score during drug administration was 0. In the group B, 70% had a VAS score of 4-6, and 30% had a VAS score of 1-3 pre-procedurally. The mean VAS score during procedure was 3.06 for group A and 1.03 for group B.

Conclusion

The study inferred that topical anesthesia is a better choice in superficial dermatosurgical procedures for providing adequate anesthesia and improved compliance when compared to infiltrative anesthesia.

## Introduction

The skin, along with its subcutaneous tissue, constitutes one of the major organ systems of the human body. Dermatosurgery is the branch dealing with skin conditions that operate at the level of the skin without disturbing the interior milieu of the human organ system [1]. It has been an evolving subspecialty of dermatology over the past decade with the potential for complex procedures, but still, microsurgical methods are used for various dermatological conditions [2]. Local anesthetics are agents applied topically over the surface to be operated or used as infiltration locally to produce reversible loss of sensations, such as pain and touch, within a defined area of the procedure. Local anesthetics can be administered in a variety of techniques. Traditionally, anesthetics were injected into the area to be operated on, which by itself may lead to severe pain in some subjects. It may also change the normal anatomical relations in dermatosurgical procedures [3].

Local anesthetic agents, which were the basis of use of the modern local anesthetic agents in the field of dentistry and medicine, have evolved nowadays [4]. The skin is one of the major sensory organs by which humans and other organisms perceive the world and in turn, are sensed by it. When these sensations go awry, a great distress may occur. When the skin is affected by any dermatologic morbidity, psychological sequelae in the form of another comorbidity often follow, which would greatly impact the patient’s quality of life [5]. Some topical anesthetic creams such as lignocaine, prilocaine, and tetracaine have evolved drastically into amalgams that improve compliance and prolong the duration of the anesthesia. To elevate the potency and efficacy of local anesthetic agents, augment the depth of the local anesthetic agent, and extend the duration of topical anesthetic drugs, varied amalgams have been researched recently and used variedly. Several studies are ongoing to collate the efficacy of different amalgams of topical anesthetics in different dermatological and aesthetic procedures [5].

## Materials and methods

The study was conducted in the Outpatient Department of Dermatology, Venereology, and Leprosy of Karpaga Vinayaga Institute of Medical Sciences and Research Centre in Chengalpattu, Tamilnadu, India. This cross-sectional study included two groups with a total of 100 patients, with 50 patients in each group (A and B). Participants were allocated alternatively into groups A and B, such that the first patient was allocated to group A, the second patient to group B, and so on.

For group A, the topical anesthetic agent used was a eutectic mixture of 2.5% lignocaine and 2.5% prilocaine cream (EMLA), specifically Prilox cream (Neon Laboratories Limited, Mumbai, India). Under sterile precautions, the cream was applied as a thick film (1-2 mm thick) over the area for the procedure and covered with a thin occlusive and adhesive sheet provided within the topical anesthetic package. After 40-45 minutes of application, the EMLA cream was removed with a wet saline-soaked gauze, and the areas were cleansed and sterilized with the povidone iodine and spirit before the procedure.

For group B, 2% lignocaine injection, specifically Lox 2% (Neon Laboratories Limited, Mumbai, India), was used as a local or regional infiltration anesthetic agent. A test dose was given over the left forearm 5 cm below the cubital fossa, and a 15-minute waiting time was allotted before the start of the procedure. If no allergic reaction was observed with the infiltration agent, the area was cleansed and draped under aseptic conditions, after acquiring informed and written consent from the patient.

Patients were thoroughly screened, a comprehensive history was obtained, and the patients were examined clinically for study selection. The subjects fulfilling the inclusion criteria were included in the study after acquiring informed and written consent. All participants were requested to rate the pain perception during anesthetic drug administration and during the surgical procedure using a visual analogue scale (VAS) separately. The immediate post-procedure pain score was recorded using the VAS system on a scale of 1-10. Results were recorded using a preformed proforma, which included a brief questionnaire for every subject and compiled. The recorded results were analyzed by a different investigator to avoid bias, then the conclusions were drawn.

Patients were examined for the local side effects during and post-procedure, recorded in the individual subject proforma. Duration of the pain while administering anesthetic agent and after the procedure were asked from individual patients and recorded separately. The mean duration of the anesthetic effect, pain due to the anesthetic agent infiltration, and post-procedure pain were also recorded and compiled. The patients requiring the intervention for after-effects of the procedure were taken care of when necessary. Quantitative variables were described as mean and standard deviation, and the qualitative variables were described as percentages. A t-test was used to find the association between the groups. All the data was analyzed using SPSS software version 23 (IBM, Chicago, USA) and results were drawn.

Study participants included were subjects above the age of 18 years. Among them, the participants included were those who understood the study protocol and were willing to give participation consent. Only the participants who were able to rate the pain and those willing to follow the study protocol were included. This study protocol took into consideration dermatological conditions that warrant the need for superficial dermatosurgical procedures only.

Pediatric patients and participants who were unable to rate the pain according to VAS score were excluded from the study. Subjects who were not willing to give consent for the procedure were excluded from the study group. Patients with a history of allergy to anesthetic agents, psychiatric illness, or neurological diseases or disorders consistent with altered sensations and Hansen's disease were excluded after obtaining the details and clinical examination. Pregnant females and lactating mothers were also excluded from the study.

## Results

A total of 100 patients attending the dermatology outpatient department for dermatosurgical procedures were examined and included in the study after fulfilling the inclusion and exclusion criteria.

The most common age group among the subjects was 21-40 years (50%), followed by < 20 years (25.6%). Males were 57.8% and females were 42.2%. Both groups had similarities with respect to age and gender. The P value was 0.143, with significance value > 0.05).

In this study, we included various dermatosurgical procedures in which local anesthetics were used. Among the subjects presented with various dermatoses, the highest number of patients had alopecia at 22%, and the lowest number had trachyonychia at 5% and acne scars at 5% (Table 1).

**Table 1:** Dermatoses for which the local anesthesia was administered LSC: Lichen simplex chronicus; MC: Molluscum contagiosum; LEN: Linear epidermal naevus; n: Number of dermatoses

Dermatoses	Topical anesthetic cream		Infiltration anesthesia		Total	
					n=100	
	n	%	n	%	n	%
Acne scar	3	6	2	4	5	5
Alopecia (areata, androgenic)	12	24	10	20	22	22
Seborrheic keratosis and its variants	5	10	14	28	19	19
Colloid milium/milia	5	10	2	4	7	7
Chronic eczema (LSC)	4	8	4	8	8	8
Infections (MC, Verruca)	10	20	8	16	18	18
Naevus (compound/LEN)	4	8	2	24	6	6
Pyogenic granuloma	0	0	7	14	7	7
Striae distensae	3	6	0	0	3	3
Trachyonychia	4	5	1	2	5	5
Total	50	100	50	100	100	100

We used local anesthetics in a variety of procedures, of which the majority number constituted electrocautery (37%) followed by intralesional PRP (16%). The least number belonged to intralesional steroid with cryotherapy (2%) (Table 2).

**Table 2:** Distribution of dermatosurgical procedures among the groups PRP: Platelet-rich plasma; n: Number of procedures

Dermatosurgical procedure	Topical anesthetic cream			Infiltration anesthesia		Total	
	n	%		n	%	n	%
Electrocautery	12	24		25	50	37	37
Extirpation	4	8		0	0	4	4
Intralesional bleomycin	2	4		2	4	4	4
Intralesional PRP	14	28		2	4	16	16
Microneedling and PRP	10	20		2	4	12	12
Intralesional steroid injection	2	4		3	6	7	7
Intralesional steroid with cryotherapy	1	2		1	2	2	2
Nail avulsion	0	0		10	20	10	10
Radiofrequency ablation	2	4		3	6	5	5
Shave excision	1	2		2	4	3	3
Total	50		100	50	100	100	100

The mean procedure duration for groups A and B was 7.30 ± 4.20 minutes and 7.77 ± 4.04 minutes, respectively. The mean procedural time was similar in both groups with a P value of > 0.05 (Table 3).

**Table 3:** Comparison of mean procedure duration between the groups n: number of procedures P value > 0.05: statistically not significant

Groups	n	Mean duration (minutes)	Standard deviation	F value	P value
Topical anesthetic cream	50	7.30	4.20	0.141	0.869
Infiltration anesthesia	50	7.77	4.04		

In group A, the VAS score during drug administration was 0. In group B, 70% had a VAS score of 4-6, while 30% had a VAS score of 1-3 pre-procedure. The mean VAS score while performing the procedure was 3.36 for group A and 1.33 for group B (Table 4).

**Table 4:** Distribution of VAS score during drug administration between the groups VAS: Visual analog scale; n: Number of subjects

VAS score	Topical anesthetic cream		Infiltration anesthesia	
	n	%	n	%
0	50	100	0	0
1-3	0	0	15	30
4-6	0	0	35	70
>6	0	0	0	0

The mean VAS score during drug administration was 0 in group A and 3.83 in group B. During the procedure, the mean VAS score for group A is 3.36 and 1.33 for group B. The mean VAS score during the procedure for group A was higher when compared to group B. The above pattern was found to be significant statistically with a P value of 0.001 (< 0.05) (Table 5).

**Table 5:** Comparison of mean VAS score during procedure between the groups VAS: Visual analogue scale; n: Number of procedures *P value < 0.05: statistically significant

Groups	n	Mean	Standard deviation	F value	P value
Topical anesthetic cream	50	3.06	1.21	43.74	0.001*
Lignocaine infiltration	50	1.03	0.84		

Post-procedure mean VAS score is 2.8 for group A and 1.0 for group B. The post-procedural VAS score was comparable to the procedural VAS score, with the VAS score higher for group A when compared with group B (Table 6). The P value was 0.0026 (< 0.05), which was statistically significant.

**Table 6:** Post-procedural VAS score VAS: Visual analog scale; n: Number of procedures

Group	n	Mean VAS score
Topical anesthetic cream	50	2.8
Lignocaine infiltration	50	1.0

In this study, the mean VAS score according to the site was lower in the face (1.32) and neck (1.78) compared to other sites. In group A, the VAS score was lower in the neck (2) and face (2.14). In group B, the VAS score was lower in the lower limb (0.67) followed by the face (1) (Table 7).

**Table 7:** Comparison of VAS scores according to site between the groups VAS: Visual analog scale

Site	Topical anesthetic cream (mean VAS score)	Infiltration anesthesia (mean VAS score)	Mean VAS score
Face	2.14	1	1.32
Neck	2	1.33	1.78
Trunk	3.33	1.67	2.05
Upper limb	4.17	1.25	2.01
Lower limb	3	0.67	2.22
Scalp	3.42	2.25	2.33

The mean duration of pain relief was 127.66 ± 88.14 minutes (minimum: 20 minutes; maximum: 300 minutes) for group A, while 72.16 ± 60.15 minutes (minimum: 20 minutes, maximum: 240 minutes) for group B. The mean duration for the pain relief was greater in group A than in group B. The above difference in the mean duration of pain relief was statistically significant with a P value of 0.001 (< 0.05) (Table 8).

**Table 8:** Comparison of mean duration of pain relief between the groups n: Number of procedures ^*^P value < 0.05: statistically significant

Groups	n	Mean duration (minutes)	Standard deviation	F value	P value
Topical anesthetic cream	50	127.66	88.14	17.89	0.001^*^
Lignocaine infiltration	50	72.16	60.15		

In this study, around 84.5% did not show any adverse events. Among group A, 6.7% had mild edema and 6.7% had mild itching at the site of application, which was short-lived. In group B, 6.7% had edema and 3.3% had very mild itching. All groups were found to be similar with respect to adverse events, with a P value of 0.314 (> 0.05).

The VAS scores during drug administration or pre-procedural, procedural, and post-procedural scores were also recorded in the patients. These values were subject to individual variations.

## Discussion

In this study, the majority of the participants were in the age group of 20-40 years, accounting for 50% of the total subjects. The mean age group of the study population was 30.9. This was almost near to the age distribution of 35.4 years seen in the study done by Friedman et al [6]. This showed that the adolescent age group was more cosmetically concerned and willing to undergo dermatosurgical procedures compared with other age groups.

This study depicted a slight increase in the number of male subjects (57%) compared to female participants (43%). This observation did not collate with other studies in which female participants were the predominant group. This pattern suggested that both male and female participants were concerned aesthetically, with more male subjects preferring surgical modality of treatments, whereas their female counterparts preferring more conservative treatment.

Among the distribution of dermatoses of 100 patients, a predominant number had alopecia (22%) followed by seborrheic keratoses and its variant (19%), viral infections such as molluscum contagiosum and warts (18%), and chronic eczema (8%). The least number had acne scars (5%). This was similar to the study conducted by Gupta et al. [7] and Croxtall [8] in which they conducted the study with 120 participants, with variable dermatoses such as alopecia, warts, milia, keloid, acrochordon, vitiligo, and intradermal nevus.

In this study, the waiting period for infiltration anesthesia was 5 minutes compared to 40-45 minutes for topical anesthetic cream. Infiltration anesthetic agents act more rapidly than topical creams, which take time to penetrate the skin layers. Various studies have different cut-offs for the waiting period. The study by Carter et al. suggested a waiting time of 30 minutes for topical anesthetic cream for superficial procedures, which is almost similar to this study [9]. The mean duration of the procedure in this study in various groups was around 7.27 +/- 1.99 minutes. It was close to the study by Gahalaut et al., in which the duration of procedures was 5-7 minutes on average [3].

In group A, the pre-procedural VAS score for pain during drug administration was 0, whereas the mean VAS score for pain was 3.83 for group B. Out of the total procedures performed under infiltration anesthesia, 70% had a VAS score of 4-6 while 30% had a VAS score of 1-3 [10].

The mean VAS score for pain during the procedure was 3.36 for group A and 1.33 for group B. The mean VAS score for pain during the procedure for group A was higher compared to group B. It was similar to the study by Alster et al. [11]. It was also similar to the studies by Giordano et al. [12] and Bourne et al. [13] in which they found that topical anesthetic cream provided similar analgesia compared to injectable lignocaine.

In this study, the significantly peaked VAS severity scores with EMLA cream might be due to the shorter duration of application. Topical anesthetic agents do not provide adequate anesthesia at the periphery. The depth of analgesia achieved was 3 mm after 60 minutes and 5 mm after 120 minutes of application. The duration of application to obtain adequate anesthesia exceeded 60 minutes, which was a limiting factor for the practical use of the topical anesthetic agent in a hectic clinical setting.

The mean duration of pain relief was 127.66 minutes (40-210 minutes) for group A and 72.16 minutes (12-130 minutes) for group B. The mean duration of pain relief was higher in group A than in group B. It was similar to the study findings by Kang and Jun [14].

Regarding the need for additional requirements of anesthetic modality, 93.3% of group A and 100% of group B tolerated the procedure well and did not require any intervention. It was similar to the study by Kilmer et al. in which 95% of the patients tolerated the procedure without additional anesthetic requirements [15]. These findings were subject to individual variations, type, and depth of the procedure performed. It inferred that most of the common superficial aesthetic procedures can be performed with topical anesthetics alone with less discomfort for the patients.

Lignocaine infiltration has a faster onset (1-5 minutes) and shorter duration of action (60-70 minutes). Lignocaine, when combined with other anesthetics such as prilocaine (120-240 minutes) or tetracaine (180-600 minutes), has a longer duration of action. The onset of action and duration depend on the pKa and protein binding of the drug. Lignocaine and prilocaine with a low pKa (7.9) are faster-acting than tetracaine with a higher pKa (8.5). Tetracaine, being more lipophilic and more protein binding (76%), is slowly released from the epidermis and has a comparatively longer duration of action than prilocaine (55% protein binding) [16].

In the side effect profile, most of the patients did not show any adverse effects though a few had transient and self-limiting reactions. In group A, two had edema (6.7%) and two had itching (6.7%). In group B, two had edema (6.7%) and one had itching (3.3%). The side effects were resolved without intervention. It was similar to the study by Gahalaut et al. [3].

Limitations of this study

The sample size of this study was relatively small, and there is a necessity for a large group study to include more spectrum of dermatosurgical cases. Since pain is a subjective phenomenon, perception may vary between individuals, and there were no standard validating tools to quantify it. This study is single-blinded and prone to bias, which needs additional research. The potential gaps in the literature of this field have to be addressed by further larger studies. Patients were allocated into groups alternatively, and no randomization was done during allocation.

## Conclusions

The results of this study favored topical anesthetic cream. It is a better choice of anesthesia in superficial aesthetic and dermatosurgical procedures to provide adequate compliance during application compared with traditional infiltration anesthesia, which is invasive and painful during administration. Infiltration anesthesia is a better choice for deep procedures and without time constraints regarding the waiting period. Adverse effects encountered were minimal, transient, and self-limited without intervention. There is always a need to conduct varied research and include a higher number of cases in a large-scale study to shore up the paucity of literature in this field.
